# Draft Genome Sequences of Five *Pseudomonas fluorescens* Subclade I and II Strains, Isolated from Human Respiratory Samples

**DOI:** 10.1128/genomeA.00837-15

**Published:** 2015-07-30

**Authors:** Brittan S. Scales, John R. Erb-Downward, John J. LiPuma, Gary B. Huffnagle

**Affiliations:** a Division of Pulmonary and Critical Care Medicine, Department of Internal Medicine, University of Michigan Medical School, Ann Arbor, Michigan, USA; b Department of Microbiology and Immunology, University of Michigan Medical School, Ann Arbor, Michigan, USA; c Department of Pediatrics and Communicable Diseases, University of Michigan Medical School, Ann Arbor, Michigan, USA

## Abstract

We report the draft genomes of five *Pseudomonas fluorescens* strains, isolated from clinical samples. Phylogenetic analysis places three in subclade I and two in subclade II of the *P. fluorescens* species complex. The average G+C content and genomic size are 63% and 7.1 Mbp (subclade I) and 59.6% and 6.14 Mbp (subclade II), respectively.

## GENOME ANNOUNCEMENT

The *Pseudomonas fluorescens* species complex is the most diverse group in the *Pseudomonas* genus, containing at least 52 subspecies (1). Phylogenetic analysis using multilocus sequence analysis (MLSA) divides the *P. fluorescens* species complex into three subclades (1–6). Subclade III contains environmental strains *P. fluorescens* SBW25, A506, and SS101, and *P. synxantha* BG33R (1, 3). We previously reported the genomes of ten clinical isolates that map via MLSA to subclade III (7). The five new *P. fluorescens* clinically isolated strains reported here fall into subclades I and II. These subclades currently contain only strains isolated from environmental samples, such as *P. protegens* Pf-5 and CHA0 and *P. chlorarphis* 30-84 and O6 for subclade I (1, 3, 8) and *P. fluorescens* Pf0-1, R124, Q2-87, and Q8r1-96 for subclade II (1, 3, 9). Members of subclades I and II are almost exclusively studied for their plant growth-promoting activities, such as suppression of pathogens and production of plant growth hormones (1–3, 8, 9). The sources of subclade I and II strains, prior to this report, include the wheat rhizosphere (30-84; Q8r1-96 and Q2-87); the soil (O6; Pf-5 and Pf0-1); rhizosphere of shepherd’s purse (CHA0), and a silica cave (R124) (1, 3, 8, 9). 

Here, we report the first genome sequences of *P. fluorescens* strains, isolated from human clinical samples that map to subclades I and II. All were isolated from cystic fibrosis sputum. Subclade I strains were isolated between August 2006 and April 2010 from Hartford, CT, USA, and Austin, TX, USA. Subclade II strains were collected in April 2004 and April 2006 from two samples from St. Louis, MO, USA, and Omaha, NE, USA. Isolates were banked at −80°C. The 16S rRNA gene was amplified with the universal primer set 8F and 1492R, sequenced using an ABI 3730XL sequencer, and identified as *P. fluorescens* using NCBI BLASTn (10). The isolates were grown aerobically overnight in Luria broth at 34°C. Genomic DNA was isolated with the Qiagen DNeasy blood and tissue kit (catalog no. 69506). Sequence data were generated with a 100-bp paired-end library on the Illumina HiSeq 2000 platform and reads were *de novo* assembled using the DNAstar SeqMan NGen version 12 software. The subclade I genomes were assembled into an average of 93 contigs (range 44 to 137), and the subclade II genomes were assembled into 56 and 67 contigs. The Mauve aligner was used to reorder the contigs using *P. protegens* Pf-5 and *P. fluorescens* Pf0-1 as references for subclades I and II, respectively (11). The three new subclade I genomes contain, on average, 63% G+C content (range 62.8 to 63.3%) and are 7.1 Mbp (range 6.68 to 7.28 Mbp). The two new subclade II genomes contain, on average, 59.6% G+C content (58.8% and 60.3%) and are 6.14 Mbp (6.08 and 6.2 Mbp). MLSA was performed using dnaE, ppsA, recA, rpoB, guaA , mutL, pyrC, and acsA, modified from the work of Loper et al. (3). Clustering and phylogenetic tree created using MAFFT (12, 13). The MLSA tree maps three of these newly sequenced strains into subclade I and two into subclade II (3, 7–9). 

### Nucleotide sequence accession numbers.

The draft genomes have been deposited at DDBJ/EMBL/GenBank under the accession numbers LCZB00000000, LCZC00000000, and LDET00000000 for subclade I isolates AU11706, AU13852, and AU20219, respectively, and under the accession numbers LCZD00000000 and LCZE00000000 for subclade II isolates AU5633 and AU11114, respectively.
